# CVN636 potentiates orthosteric agonist responses selectively at mGlu _7/7_ homodimers

**DOI:** 10.64898/2026.10.17.759826

**Published:** 2026-10-19

**Authors:** Xin Lin, Colleen M. Niswender, Wesley B. Asher, Jonathan A. Javitch

## Abstract

The metabotropic glutamate receptor 7 (mGlu _7_ ) is an emerging therapeutic target for central nervous system disorders, but progress has been limited by a lack of selective pharmacological tools. CVN636 was recently reported as a potent and selective allosteric agonist of this receptor, capable of receptor activation in the absence of glutamate, with demonstrated efficacy in a rodent model of alcohol use disorder. Here, using complementary bioluminescence resonance energy transfer-based assays that measure both downstream signaling and G protein recruitment, we show that CVN636 does not activate the receptor on its own. Instead, CVN636 acts as a positive allosteric modulator that enhances the potency and efficacy of orthosteric agonist responses, including those of glutamate, in an agonist-dependent manner. Blockade of orthosteric agonist binding with a competitive antagonist abolished CVN636 activity, consistent with this mechanism. Furthermore, using an assay that distinguishes signaling from defined receptor dimers, we find that CVN636 activity is selective for mGlu _7/7_ homodimers and does not extend to any mGlu _7_ -containing heterodimer tested. Thus, in our assay system CVN636 is an mGlu _7/7_ homodimer-selective positive allosteric modulator of this receptor, suggesting that its i *n vivo* actions likely stem from action at these homomers.

